# Monocytosis as prognostic factor for chronic graft versus host disease

**DOI:** 10.1038/s41409-024-02333-z

**Published:** 2024-06-22

**Authors:** C. Marrero-Cepeda, T. Caballero-Velazquez, S. Garcia-Canale, F. Martin-Dominguez, N. Rodriguez-Torres, I. Espigado-Tocino, C. Blazquez-Goñi, H. Andrade-Ruiz, J. Perez-Simon

**Affiliations:** 1 Department of Hematology, University Hospital Virgen del Rocio, Instituto de Biomedicina de Sevilla (IBIS), CSIC, University of Seville, Seville, Spain; 2 FISEVI, University Hospital Virgen del Rocio, Seville, Spain

**Keywords:** Risk factors, Haematological cancer

## To the Editor:

Chronic graft versus host disease (cGvHD) is the main cause of long-term morbidity and mortality after allogeneic hematopoietic stem cell transplantation (HSCT) [1].

Several biomarkers have been described which predict the risk of acute GvHD (aGvHD) and its severity. In this regard, suppressor of tumorigenicity-2 (ST2) and regenerating islet-derived protein 3-α (REG3α) predict the risk for aGvHD and GvHD-related mortality (NRM) [2] according to the Mount Sinai Acute GVHD International Consortium (MAGIC) [3, 4]. By contrast, no cGvHD prognostic biomarkers have been clearly stablished during follow up.

Finding a prognostic biomarker for cGvHD onset is an unmet need in clinical practice. A biomarker could be used to guide prophylaxis treatments and follow-up strategies. High-risk patients could stay longer with high-dose immunosuppressive therapy, change the prophylaxis regimen to a more intensive one or targeting another pathway or mechanism of action. When identifying a biomarker, it is to take into account its sensitivity and specificity; particularly the latter in order to avoid false positive cases which might lead to non-justified treatments.

Considering the important role of monocytes in cGvHD pathophysiology, we analyzed the potential role of monocytosis as cGvHD biomarker.

We performed a retrospective study including patients who received allogeneic HSCT in our center between 2014 and 2017. We excluded patients who died within the first 100 days post-transplant. Overall, 135 patients were included into the analysis. Monocytes were analyzed from transplantation until death or last follow-up. We only considered as prognostic factor the monocytosis that appeared within 6 months before the diagnosis of cGvHD.

Cumulative incidence (CI) of cGvHD of patients at risk at 36 months was 66.9% (range 56.7–74.7%), with a CI of moderate-severe of 52.3% (range 40.7–61.6%) and severe of 29.1% (range 18.5–38.4%). Overall survival (OS) at 36 months was 60.7% (range 51–72.2%) and the CI of relapse at 36 months was 36.3% (range 24.6–46.2%).

**Fig. 1:**
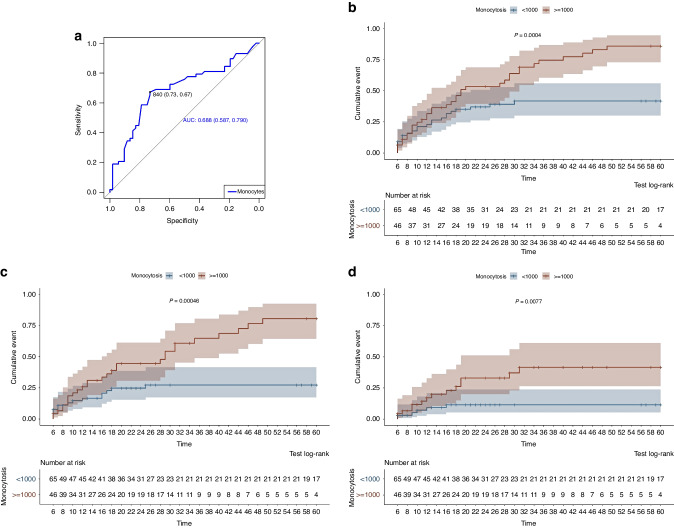
Monocytosis as prognostic factor for cGvHD: ROC curve and cumulative incidence (CI). **a** ROC curve. The most predictive value was 0.84 × 10^9^/L monocytes, with a sensitivity of 73% and a specificity of 67%. Cut-off of 1.0 × 10^9^/L monocytes was also an optimal value with a sensitivity of 59.3% and a specificity of 78.8%. **b** CI of overall cGvHD at ≥6 months after alloHSCT among patients with more or less than 1.0 × 10^9^/L monocytes. **c** CI of moderate-severe cGvHD and **d** CI of severe cGvHD, also among patients with more or less than 1.0 × 10^9^/L monocytes.

Interestingly, post-transplant monocytes count highly correlated with the risk of cGvHD. The most significant results were observed at ≥6 months post-transplantation. Median monocyte count was 1.0 ×10^9^ / L among patients who subsequently developed cGvHD as compared to 0.695 ×10^9^ / L among those who did not (*p* = 0.00067). The most prognostic cut-off value in the ROC curve was ≥0.84 ×10^9^/L, but a cut-off value of ≥1.0 ×10^9^/L was also highly prognostic and, therefore, it was used in the study (Fig. 1a). With this cut-off value at ≥6 months post-transplantation, the risk of cGvHD at 36 months was 74.6% (range: 56.6–85.19%) vs 41.8% (range: 27.1–53.5%) for patients with more or less than 1.0 ×10^9^/L monocytes (*p* = 0.0004) (Fig. 1b). cGvHD flared at a median of 19 months in patients with >1.0 ×10^9^/L monocytes and it was not reached during follow-up in patients with lower counts (*p* = 0.0004).

Moreover, monocyte count was highly prognostic not only for the incidence but also for cGvHD severity: CI of moderate-severe cGvHD at 36 months were 64.7% (range 43.9–77.82%) vs 27.3% (14.2–38.4%), among patients with and without monocytosis, respectively (*p* = 0.00046) (Fig. 1c) while these values for severe cGvHD were 41.6% (range 21–56.9%) vs 11.4% (range 2.3–19.7%) (*p* = 0.0077) (Fig. 1d).

Finally, we performed a multivariate analysis for overall cGvHD including monocytes count, GvHD prophylaxis, donor type, HLA matched and prior aGvHD. Patients with ≥1.0 ×10^9^/L monocytes during follow-up had a HR to develop cGvHD 2.1 times higher than those with a lower count (*p* = 0.006). Similarly, the risk to develop moderate-severe cGvHD was 2.93 times higher among patients with ≥1.0 ×109/L monocytes as compared to those with lower counts (*p* = 0.001).

A competing risks model was performed to further evaluate the impact of monocytes count on the incidence of overall cGvHD: 66.99% vs 36.53% for patients with or without monocytes >1.0 ×10^9^ / L developed it. Using a Fine-Gray regression, patients with monocytosis had 2.39 greater risk of developing overall cGvHD (*p* = 0.0029).

Reviews by Crossland et al. and Giaccone et al. analyzed immune cells, alloantibodies, glycans, microRNA, extracellular vesicles, endothelial-derived microparticles, DNA methylation and microbiome changes as potential cGvHD biomarkers [5, 6] CD19+ CD21− B cells are increased in patients with first diagnosis of cGvHD and they could predict higher risk of BOS [7–9]. Elevated Th17 T cells have also been reported in blood or target organs of cGvHD patients [10–14]. Furthermore, early CD8+ T-cell recovery on day 28 after HSCT could predict lower risk of relapse but higher of GvHD.

Pan et al. suggested that anti-HLA antibodies could be a prognostic biomarker for cGvHD [15]. Also, microRNA has been widely studied in aGvHD with limited evidence for cGvHD. However, a study in mice showed a miR-17–92 role in scleroderma and BOS [16] and Lacina et al. studied altered microRNA profile in extracellular vesicles in cGvHD patients three months after transplantation [17].

Chirumbolo et al. created a multiparameter prognostic risk score of cGvHD including previous aGvHD (*P* = 0.007), CXCL10 serum concentrations (*P* = 0.008) and plasmacytoid dendritic cell peripheral levels (*P* < 0.001) at 3 months after HSCT, with promising results that need to be validated [18]. As the options described above, these biomarkers are not easily applicable in clinical practice. This is also the inconvenience for biomarkers proposed by Kordelas et al.: decreased Programmed Cell Death Protein 1 (PD-1) levels [19] and low soluble HLA-E levels [20]. A more accessible biomarker in any routine lab would be prolactin and, in this regard, a study by Salas et al. found out that hyperprolactinemia was related to cGvHD activity [21].

In cGvHD pathophysiology, immune cell populations are altered, including monocytes. Stimulated monocytes produce IL-1 and TNFα. Dendritic cells are activated by TNFα leading to increased alloantigen presentation, inflammatory chemokines production, immune cells migration to affected organs and tissue damage due to apoptosis and necrosis [22, 23]. A study performed by Arpinati et al. showed that marrow and blood monocytes were increased and activated in patients with cGvHD when compared to those without (*P* = 0.006) [24]. Moreover, promising data have been reported using Axatilimab, which targets CSF-1R expressed in monocytes, among patients with cGvHD which failed previous lines of therapy [25]. CCR2+ monocytes generate monocyte-derived alveolar macrophages, leading to the development of alloimmunity and bronchiolitis obliterans syndrome, which are both involved in cGvHD [26].

Monocytes’ count would be an excellent biomarker due to its accessibility. By contrast, most biomarkers previously identified are time consuming and/or difficult to obtain [27].

Our findings suggest that monocytosis may influence clinical decisions, possibly with a positive impact in early diagnosis. The role of biomarkers in adjusting immunosuppressive therapies, prognosis and outcomes of the disease is unknown, but it is clearly a promising field of investigation. It would also be important to take into consideration other factors that might cause monocytosis in order to avoid false positives and unnecessary treatments.

In conclusion, monocytosis onset from 6 months after allo-HSCT is a highly prognostic biomarker of cGvHD. Monocytes’ count ≥1.0 10^9^/L may identify patients with higher risk of cGvHD, particularly those with moderate and severe manifestations.

Further investigations are needed to apply these findings on developing personalized approaches for cGvHD management.
